# Glycoproteomics-based signatures for tumor subtyping and clinical outcome prediction of high-grade serous ovarian cancer

**DOI:** 10.1038/s41467-020-19976-3

**Published:** 2020-12-01

**Authors:** Jianbo Pan, Yingwei Hu, Shisheng Sun, Lijun Chen, Michael Schnaubelt, David Clark, Minghui Ao, Zhen Zhang, Daniel Chan, Jiang Qian, Hui Zhang

**Affiliations:** 1 Department of Pathology, Johns Hopkins University, School of Medicine, Baltimore, MD 21287 USA; 2 Department of Ophthalmology, Johns Hopkins University, School of Medicine, Baltimore, MD 21287 USA

**Keywords:** Proteomics, Mass spectrometry, Tumour heterogeneity, Glycosylation

## Abstract

Inter-tumor heterogeneity is a result of genomic, transcriptional, translational, and post-translational molecular features. To investigate the roles of protein glycosylation in the heterogeneity of high-grade serous ovarian carcinoma (HGSC), we perform mass spectrometry-based glycoproteomic characterization of 119 TCGA HGSC tissues. Cluster analysis of intact glycoproteomic profiles delineates 3 major tumor clusters and 5 groups of intact glycopeptides. It also shows a strong relationship between N-glycan structures and tumor molecular subtypes, one example of which being the association of fucosylation with mesenchymal subtype. Further survival analysis reveals that intact glycopeptide signatures of mesenchymal subtype are associated with a poor clinical outcome of HGSC. In addition, we study the expression of mRNAs, proteins, glycosites, and intact glycopeptides, as well as the expression levels of glycosylation enzymes involved in glycoprotein biosynthesis pathways in each tumor. The results show that glycoprotein levels are mainly controlled by the expression of their individual proteins, and, furthermore, that the glycoprotein-modifying glycans correspond to the protein levels of glycosylation enzymes. The variation in glycan types further shows coordination to the tumor heterogeneity. Deeper understanding of the glycosylation process and glycosylation production in different subtypes of HGSC may provide important clues for precision medicine and tumor-targeted therapy.

## Abstract (web summary)

Altered protein glycosylation is increasingly recognized as a hallmark of cancer. Here, the authors profile the glycoproteome of 119 high-grade serous ovarian carcinoma tissues, showing that glycosylation patterns correlate with tumor molecular subtypes and clinical outcomes.

## Introduction

As the most common and aggressive type of ovarian cancer, high-grade serous ovarian carcinoma (HGSC) remains the leading cause of ovarian cancer-related death^1,2^. Advanced-stage HGSC is representative of >50% of all ovarian carcinomas, and patients are shown to have a poor prognosis: 32.1% for 5-year survival and 15% for 10-year survival^3^. The complexity of HGSC is enhanced by tumor heterogeneity, which can be divided into inter-tumor and intra-tumor heterogeneity. Intra-tumor heterogeneity has a crucial role in metastasis, treatment, recurrence, and therapeutic resistance, while inter-tumor heterogeneity as well as surgical procedures and genomic instability could cause variation in survival rates within HGSC^4–9^. Several large-scale studies have provided perspectives that integrated the aberrations in HGSC with extensive inter-tumor heterogeneity, as well as evidence that HGSCs display disparate molecular profiles, which involves genomic, transcriptomic, proteomic and phosphoproteomic features^10,11^.

Proteoforms from different protein modifications add a tremendous amount of complexity to cellular proteomes^12^. Glycosylation is one of the most common types of protein modifications^13^. Distinct from the regulatory elements of gene expression and protein sequencing controlled by a DNA-template and mRNA translation, respectively, protein glycosylation is controlled by the expression and activity levels of glycotransferases and glycosidases that are involved in the glycosylation biosynthesis process^14^. The structures of glycoconjugated glycans reflect tightly regulated enzymatic biosynthetic reactions, and give rise to a variety of glycan distributions^15^. Glycoconjugates mediate the characteristics of cell surfaces and add a level of plasticity to the functions of the genetically coded protein gene products. Recently, glycosylation has been added as a new hallmark of cancer^16,17^. It plays a major role in carcinogenesis, including cell–matrix interaction, cell signaling and communication, tumor angiogenesis, immune modulation, and tumor metastasis^18,19^. Targeting glycosylation has become a potential therapeutic approach^20^. Inhibiting the ER α-glucosidases at a low level could disrupt the folding of these proteins, and potentially be of therapeutic use in treating viral infections, without affecting host-cell viability^21^. Moreover, glycosylation increases tumor heterogeneity because aberrant glycan modifications are cell-specific, protein-specific, and site-specific^19^.

Although great progress has been made in understanding the cancer genome, proteome, and phosphoproteome, there is still a relative lack of understanding when it comes to the larger glycoproteome of ovarian cancer. Previous studies have examined aberrant glycosylation in ovarian tumors or in sera of patients with ovarian cancer^22–24^. Further investigation is needed to address the roles of glycotransferases and glycosidases on glycan structures, how the degree of glycan structure changes is related to inter-tumor heterogeneity, and the clinical implications of these glycosylation features. A systems biology approach that integrates multi-omics data sets is a useful approach for addressing these questions^11,24–27^, and has great potential for linking genomic alterations to glyco-phenotypes.

In this study, we performed mass spectrometry (MS)-based glycoproteomics analysis on 119 HGSCs. These HGSC tumor tissues have been previously characterized at the genomic and transcriptomic levels, as well as at the proteomic and phosphoproteomic levels, by The Cancer Genomic Atlas (TCGA) consortia and the Clinical Proteomic Tumor Analysis Consortium (CPTAC)^10,11^, respectively. Employing two glycoproteomic strategies, solid-phase extraction of glycosite-containing peptides (SPEG) for glycosite analysis^28^, and intact glycopeptides for investigation of glycosite-specific glycans (IGPs)^29,30^, we profile the N-linked glycoproteome in HGSCs, identifying and quantifying glycosites and their attached glycan structures. Through the integration of genomics, transcriptomics, proteomics, and glycoproteomics, we identify glycosylation changes associated with distinct HGSC subtypes, and further examine the roles of substrate proteins and glycosylation enzymes in the biosynthesis of glycoproteins. Moreover, integrating clinical information facilitates the identification of glycoproteomics-based signatures associated with patient survival. Overall, this study reveals the potential roles of glycosylation in ovarian cancer heterogeneity, and provides rationale for patient stratification based on glycoprotein expression patterns.

## Results

### Identification and quantification of glycopeptides

A total of 169 TCGA ovarian tumors from HGSC were analyzed by JHU and PNNL^11^. The JHU team analyzed 119 available ovarian tumors, which were further analyzed using glycoproteomics in this study (Supplementary Data 1). Tumor tissues were lysed and proteins were extracted, which were then followed by proteolytic digestion and subsequent labeling of peptides with isobaric tags for relative and absolute quantitation (iTRAQ). Independently, a quality control (QC) sample was included in the multiple repeated analyses for longitudinal assessment of reproducibility of the sample processing and data acquisition pipelines. The iTRAQ-labeled peptides were split into three aliquots for global proteomic analysis and the parallel enrichments of glycopeptides for glycosite-containing peptides from SPEGs and intact glycopeptides (IGPs)^28,30^. The enriched glycosite-containing peptides and IGPs were analyzed by LC–MS/MS and subsequent data analysis (see “Methods” section, Supplementary Fig. 1). As described in Supplementary Fig. 1A, the SPEGs and IGPs were enriched from the same iTRAQ-labeled peptides from CPTAC Global proteomic analysis^11^. This allowed direct comparison of SPEG, IGP, and global proteins (see “Methods” section, Supplementary Fig. 1B).

In total, 5115 N-linked SPEGs and 15,512 N-linked IGPs were identified in the SPEG and IGP experiments, respectively (Supplementary Data 2), with 455 SPEGs and 351 IGPs quantified across all tumor samples. Among those peptides, 4957 (96.9%) of the 5115 SPEG glycopeptides and 14,264 (92.0%) of the 15,513 intact glycopeptides had the glyco NxS/T motifs. Prior to subsequent data analysis, SPEG and IGP abundances were normalized by the median value of global glycoproteins for each sample. In addition, we utilized our QC channel to assess the overall data quality and determine the reproducibility of the glycoproteomic analysis using coefficient of variation (CV) of individual peptides (Supplementary Fig. 2). The reported median CVs (inter-quartile ranges) were 7.0% (4.0%) and 14.0% (8.0%) for SPEGs and IGPs, respectively. Values were found to be much lower than those in different tumor samples (Supplementary Fig. 2A and B). Together, these indicate the high degree of reproducibility of glycopeptide enrichment and subsequent data acquisition, as well as the high overall quality of our dataset.

### Glycoproteomics-based clustering of HGSC

**Fig. 1:**
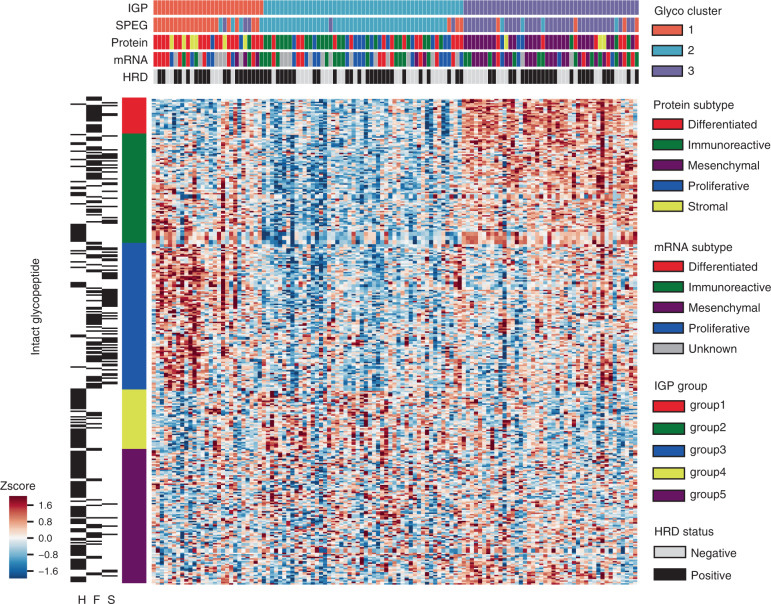
Clustering analysis of high-grade serous ovarian carcinoma based on glycoproteomics data. Bi-clustering of intact glycopeptide expression in 119 tumor tissues. Tumors are displayed as columns, grouped by intact glycopeptide clusters as indicated by different colors. Intact glycopeptides used for the tumor classification are displayed as rows with glycans shown in the right heatmap. Color of each cell indicates *Z* score (log2 of relative abundance scaled by intact glycopeptide standard deviations) of the intact glycopeptide in that sample. Transcriptome, proteomic-based subtypes, HRD annotations and the proposed SPEG, IGP clusters are indicated in color above the heatmap.

**Fig. 2:**
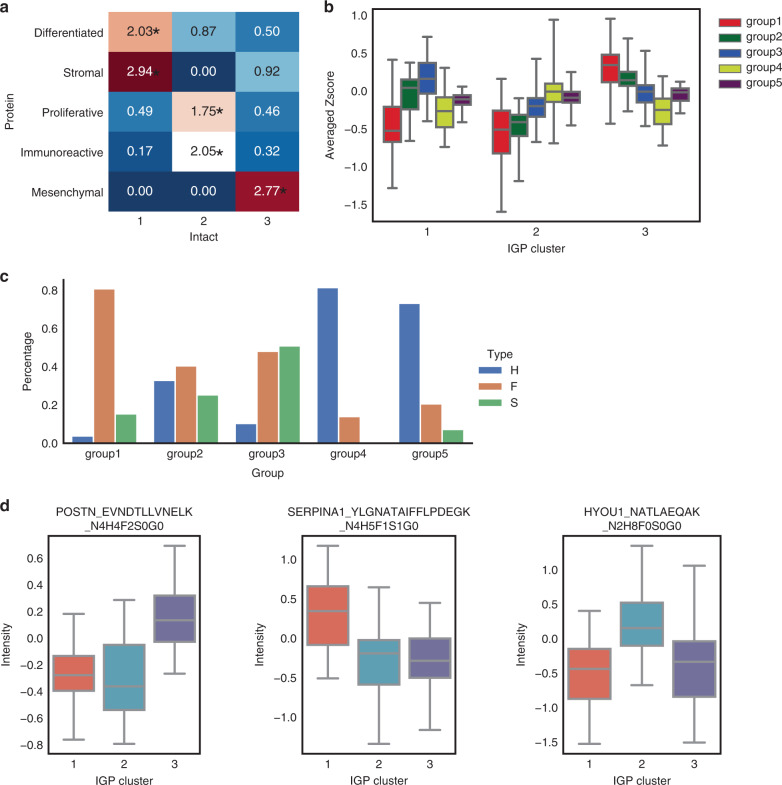
Specificity of intact glycopeptides and glycans in tumor clusters. **a** Correspondence of intact glycopeptides and proteomics clusters. Values correspond to the square of number of subjects for each proteomic subtype that belong to a corresponding IGP cluster divided by both sample number of the proteomic subtype and sample number of the IGP cluster. * Indicates a *p* value <0.01 based on a hypergeometric test. **b** Averaged *Z* score of each group of intact glycopeptides. For every group in IGP cluster 1, *n* = 27 samples; for every group in IGP cluster 2, *n* = 49 samples; for every group in IGP cluster 3, *n* = 43 samples. The box outlines denote the IQR, the solid line in the box denotes median “averaged *Z* score”, and the whiskers outside of the box extend to the minimum and maximum “averaged *Z* score”. **c** The glycans for each IGP group. **d** Three examples show the different expressions in different tumor clusters. In IGP cluster 1, *n* = 27 samples; in IGP cluster 2, *n* = 49 samples; in IGP cluster 3, *n* = 43 samples. The box outlines denote the IQR, the solid line in the box denotes median intensity, and the whiskers outside of the box extend to the minimum and maximum intensities. Source data are provided as a Source data file.

To investigate tumor heterogeneity, we leveraged the expression data of SPEGs and IGPs to cluster the 119 HGSC samples. The consensus clustering results illustrated that three tumor clusters and five glycopeptide groups could be identified based on glycoproteomics data from IGPs (Fig. 1, Supplementary Data 3). In addition, we observed a high degree of concordance when utilizing the expression patterns of SPEGs for clustering analysis. We then integrated the previously annotated transcriptomic-based and proteomic-based subtypes of HGSC previously described by TCGA and CPTAC, respectively (Fig. 1). Next, we evaluated the enrichment scores between the different omics data types to delineate the relationship between protein-based and glyco-based subtypes of HGSC, selecting a hypergeometric test *p* value of 0.01 as a threshold for overlap between different omics subtypes. As shown in Fig. 2a, the IGP 1 cluster overlapped predominately with the “Stroma” protein subtype (enrichment score = 2.94), followed by the “Differentiated” protein subtype (enrichment score = 2.03), while IGP 2 cluster overlapped with the “Immunoreactive” (enrichment score = 2.05) and “Proliferative” (enrichment score = 1.75) protein subtypes. Unique to IGP 3 was the high degree of overlap with the “Mesenchymal” protein subtype (enrichment score = 2.77) (Figs. 1 and 2a). Among the 119 HGSCs, 67 homologous recombination deficiency (HRD) samples were identified, which are annotated in Fig. 1a. Interestingly, we found there was a higher frequency of HRD samples in IGP 1 (67%), but lower frequencies in IGP 2 and 3 (~53%) (*t* test, *p* = 0.07).

Next, to identify the defining IGP signatures for each tumor IGP cluster, we averaged the *Z*-score values of individual IGPs from each of the five IGP groups (Fig. 2b). IGPs in groups 3, 4, and 1 were highly expressed in tumor clusters 1, 2, and 3, respectively (Fig. 2b). To further investigate the glycan compositions of these three IGP clusters, the percentage of three glycan types (i.e. high mannose, fucose, and sialic acid) carried by IGPs were calculated for each group (Fig. 2c). IGPs in group 1 were primarily composed of glycans containing a fucose, while IGPs in group 3 had glycans that included both fucose and sialic acid residues. IGPs in group 4 were distinct from those in groups 1 and 3, and were comprised primarily of high mannose glycans. In the downstream analysis, we focused on these three intact glycopeptide groups, i.e. groups 1, 3, and 4, with the highest expressions in corresponding clusters and clear glycan patterns (Fig. 2b and c). Specific intact glycopeptides for each IGP were provided as examples in Fig. 2d. EVNDTLLVNELK-carrying fucose from the *POSTN* protein, YLGNATAIFFLPDEGK-carrying fucose and sialic acid from the *SERPINA1* protein, and NATLAEQAK-carrying high mannose from the *HYOU1* protein were upregulated in intact subtypes 3, 1, and 2, respectively. To gain insight into the functional relevance of these intact glycopeptide profiles, we performed a STRING protein–protein interaction network analysis (Supplementary Fig. 3)^31^. We found that the IGPs in groups 1, 3, and 4 were mapped to proteins associated with the extracellular matrix, complement and coagulation cascades, and the lysosome, respectively (adjusted *p* < 0.05).

### Survival analysis of intact glycopeptide signatures

**Fig. 3:**
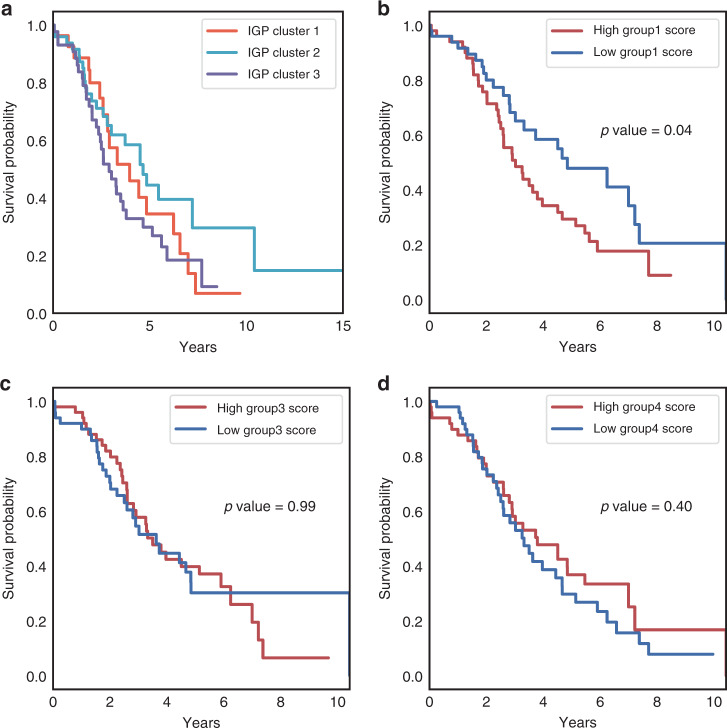
Kaplan–Meier plot of overall survival stratified by IGP clusters or IGP group signatures. **a** Three clusters. In IGP cluster 1, *n* = 27 samples; in IGP cluster 2, *n* = 49 samples; in IGP cluster 3, *n* = 43 samples. **b** Group 1 signature. **c** Group 3 signature. **d** Group 4 signature. In **b**–**d**, for both groups with highest scores and lowest scores, *n* = 50 samples. Logrank_test without adjustment is used in **b**–**d**.

To investigate whether the glycoproteomic clusters were related to clinical outcomes, we first performed survival analysis for the three clusters defined by the differential expression of IGPs (Fig. 3a). A Kaplan–Meier plot showed that cluster 3 had the worst 5-year survival prognosis, while cluster 2 had the best one. To further investigate whether the intact glycopeptides-based signatures could help with survival prediction, we calculated the values of each sample based on averaged *Z*-scores of three intact glycopeptide groups (groups 1, 3, and 4). We then selected 50 higher score samples and 50 lower score samples from each IGP group. The results showed that group 1 had a good prediction value relative to the other two groups (Fig. 3b–d), and tumors categorized as having a high group 1 score were shown to have a worse survival prognosis (Fig. 3b, *p* < 0.05). Taken together, these results indicated that the glycoproteins carrying non-sialylated fucosylated glycans were associated with HGSC tumors with worse survival outcomes. The tumor cluster with elevated levels of non-sialylated fucosylated glycans mainly contained the messenchymal HGSC subtypes from the proteomic and transcriptomic subtypes (Fig. 1a). In addition, we performed the multivariate analysis for age and IGP clusters using the cox proportional hazards regression model. Age is significantly associated with survival (*p* < 0.005), while IGP clusters have no significant association with it (*p* = 0.33). Even after adjusting for these two factors, the IGP group 1 score still maintained a strong association with survival (*p* = 0.03) (Supplementary Fig. 4A). We also investigated the association of proteins and mRNAs that corresponded to the genes in IGP group 1 with survival, and the results showed that proteins (Supplementary Fig. 4B) and mRNAs (Supplementary Fig. 4C) have no significant associations with survival, while glycoproteomic level analysis showed association of IGP group 1 with survival (Fig. 3b). Similar analyses were performed for the IGP groups 3 and 4 (Supplementary Fig. 4D–I).

### Glycosylation aberration analysis of HGSCs

**Fig. 4:**
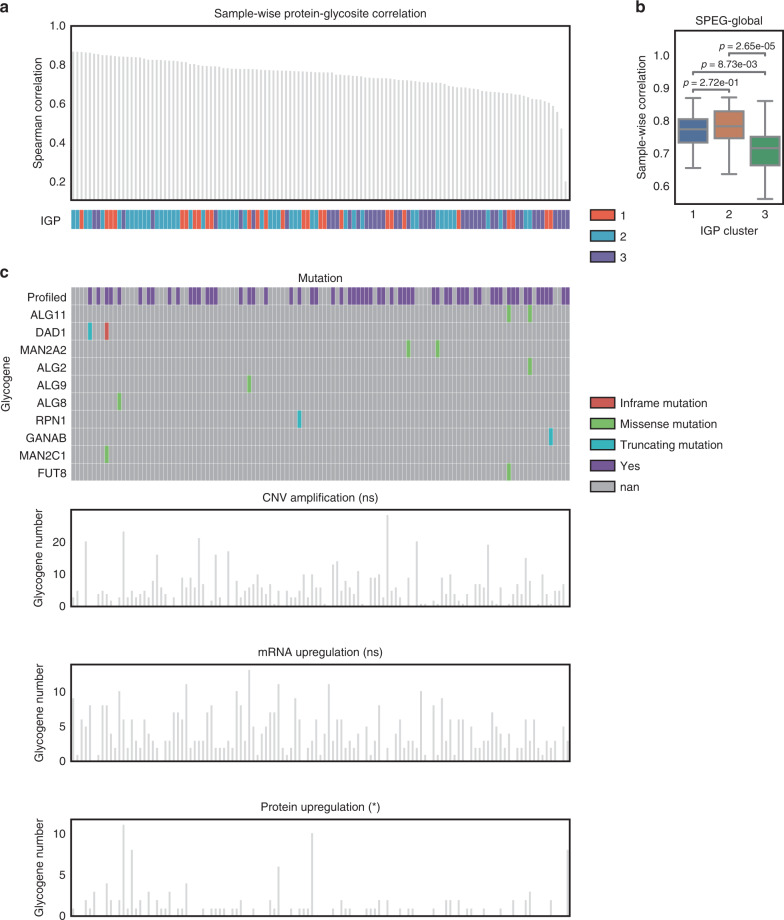
Sample-wise correlation analysis between glycosites and proteins. **a** Barplot of sample-wise correlation between glycosite-containing peptides and global proteins of 119 HGSCs. IGP clusters are shown in bottom panel. **b** Boxplot of sample-wise correlations in 3 IGP clusters. In IGP cluster 1, *n* = 27 samples; in IGP cluster 2, *n* = 49 samples; in IGP cluster 3, *n* = 43 samples. *p* = 0.272 (IGP cluster 1 vs. IGP cluster 2), *p* = 8.73e−3 (IGP cluster 1 vs. IGP cluster 3), and *p* = 2.65e−5 (IGP cluster 2 vs. IGP cluster 3) two-tailed unpaired Student’s *t*-test. The box outlines denote the IQR, the solid line in the box denotes median correlation value, and the whiskers outside of the box extend to the minimum and maximum correlation values. **c** Glyco-related gene variations at mutation, copy number amplification, mRNA, and protein levels. The number of up-regulated glyco-related gene are compared between top 50 samples with higher correlations and 50 samples with lower correlations using two-tailed unpaired Student’s *t*-test without adjustment. NS indicates non-significance, * indicates significance. Only protein level shows a significant difference (*p* = 0.03). Source data are provided as a Source data file.

Since aberrant glycosylation has been associated with oncongenesis, and is independent of DNA/mRNA gene expression profiles, we examined the degree of glycosylation aberration in HGSC by assessing the disconcordance of glycosites and total glycoprotein expression patterns. We made the hypothesis that if SPEG-global had higher correlation, i.e. low perturbation, in a sample, the glycan processing is mainly controlled by the substrate; if not correlated, the glycosylation may be regulated by other factors such as glycosylation enzymes. Sample-wise spearman correlation was calculated using SPEG data of 413 glycosite-containing peptides and global data of 120 glycoproteins. Interestingly, we observed IGP cluster 1 displayed a high correlation between global proteins and SPEGs for glycosite-containing peptides, while IGP cluster 3 displayed a lower degree of correlation (Fig. 4a, b), indicative of IGP cluster 3 samples showing a higher degree of glycosylation disturbance. To determine whether glyco-related genes were impacting the observed glycosylation disturbance, we leveraged the bioinformatic resource cBioPortal (http://cbioportal.org) in order to investigate the frequency of mutations in these respective genes^32^ (Fig. 4c). We found that mutations in glyco-related genes were quite rare, possibly reflective of a selection mechanism via cellular lethality associated with potential loss of function of these genes, or other genomic fidelity mechanisms. Copy number amplification, mRNA upregulation, and protein upregulation were also annotated for each sample in Fig. 4c. Copy number amplification and mRNA upregulation of glyco-related genes were evenly distributed among samples. In contrast, we observed, on average, 4.7 and 3.5 glyco-related genes were upregulated at the protein level among the top 50 samples with higher correlations and 50 samples with lower correlations (*t* test *p* = 0.03), respectively (Fig. 4c). Taken together, these results indicate protein-level expression of glyco-related genes has a larger impact on glycosylation disturbance compared to the impact of genomic and transcriptomics events.

### Correlation of glycopeptides to mRNA, proteins, and glycosites

**Fig. 5:**
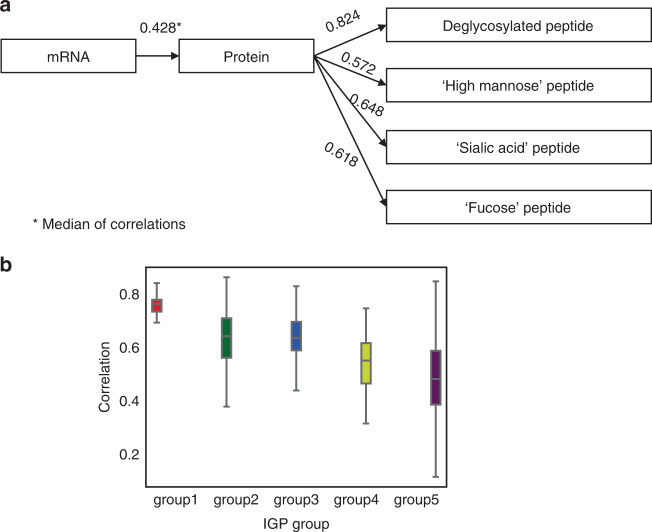
Correlation analysis between mRNA, proteins, and intact glycopeptides. **a** Correlation analysis following the central dogma. **b** Correlation analysis between proteins and intact glycopeptides in five subgroups of intact glycopeptides with different glycans. In group 1, *n* = 26 glycopeptides; In group 2, *n* = 79 glycopeptides; In group 3, *n* = 85 glycopeptides; In group 4, *n* = 43 glycopeptides; In group 5, *n* = 95 glycopeptides. For **b**, the box outlines denote the IQR, the solid line in the box denotes median correlation value, and the whiskers outside of the box extend to the minimum and maximum correlation values. Source data are provided as a Source data file.

To further expand on the assessment of the patterns of expression correlation among multi-omics, we performed gene-wise correlation of mRNA, protein, and IGP levels (Fig. 5). We found a median correlation value of 0.428 between paired mRNA transcripts and protein abundances of the identified glycoproteins, which is similar to the results reported previously in HGSCs from mRNA and overall protein levels^11^. We then evaluated the correlation between SPEGs from glycosite-containing peptides and proteins of the corresponding glycoproteins, observing a median correlation value of 0.824 (Fig. 5a). Interestingly, when we examined the correlation of different types of IGPs to global glycoproteins, we found IGPs to have a more variable expression pattern. Relative to high mannose IGPs (Spearman correlation = 0.572), complex type glycan-containing IGPs (including sialic acid and fucose glycan structures) showed a higher correlation (Fig. 5a). This trend was observed across the five IGP groups, specifically seeing a lower median correlation in groups 4 and 5 (Fig. 5b), the latter of which was enriched for high mannose glycan structures. These results posit cellular mechanisms that are aberrant or differentially regulate high mannose-containing glycan structures relative to more complex glycans.

### Correlation of glycosylation enzymes to intact glycopeptides

**Fig. 6:**
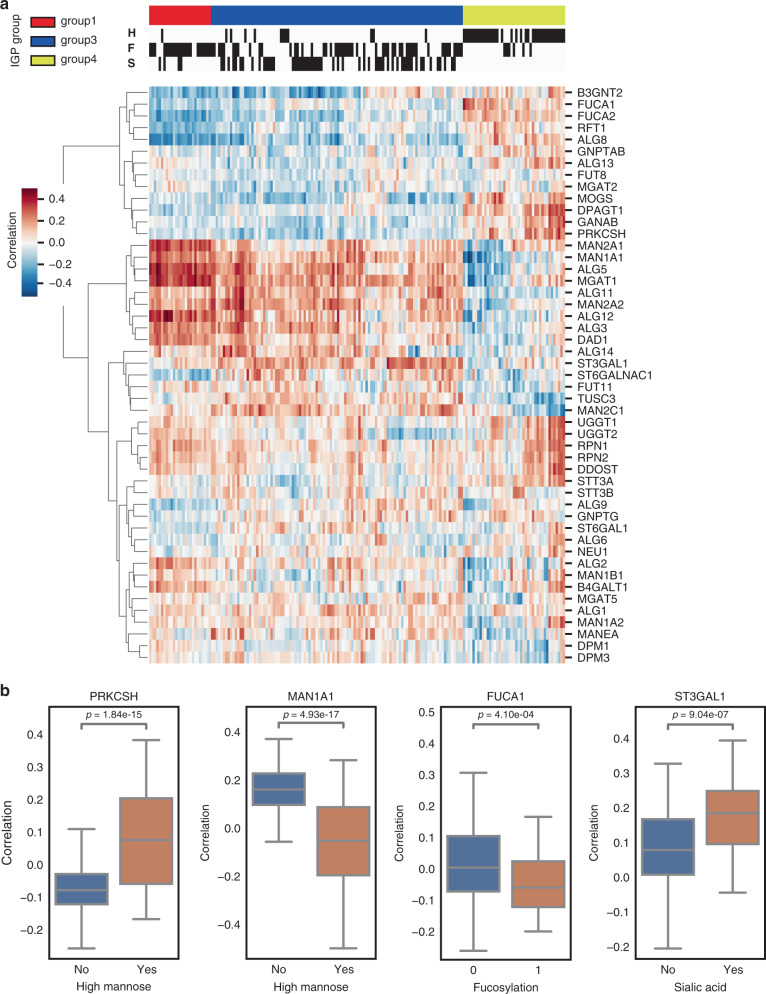
Heatmap of expression correlation between N-linked glycosylation enzyme and intact glycopeptides with different glycans. **a** Bi-clustering was performed on correlations between intact glycopeptides (column) and glycosylation enzyme expression in total protein level (row) across 119 tumor samples. Red indicates positive correlation while blue indicates negative correlation. **b** Boxplot panel of the correlation difference between enzyme expression (protein level) and specific intact glycopeptides. For PRKCSH, *n* (No) = 128 glycopeptides, *n* (Yes) = 47 glycopeptides, *p* = 1.84e-15 (Yes vs. No); For MAN1A1, *n* (No) = 128 glycopeptides, *n* (Yes) = 47 glycopeptides, *p* = 4.93e−17 (Yes vs. No); for FUCA1, *n* (No) = 97 glycopeptides, *n* (Yes) = 78 glycopeptides, *p* = 4.10e−4 (Yes vs. No); for ST3GAL1, *n* (No) = 117 glycopeptides, *n* (Yes) = 58 glycopeptides, *p* = 9.04e−7 (Yes vs. No). Two-tailed unpaired *t*-test without adjustment is used in **b**. The box outlines denote the IQR, the solid line in the box denotes median correlation value, and the whiskers outside of the box extend to the minimum and maximum correlation values. Source data are provided as a Source data file.

The conjugation and biosynthesis of *N*-linked glycans conjugated to a nascent peptide chain not only involve the glycoprotein substrates, but also a myriad of glycosylation enzymes (e.g. glycotransferases and glycosidases) to build or modify the attached glycan moiety through a series of concerted steps (Supplementary Fig. 5). To link the global protein expression of glycotransferases and glycosidases to the expression profiles of IGPs, we performed pairwise correlation analysis (Fig. 6a). We observed that two groups of enzymes were highly correlated to IGPs carrying high mannose glycans. One group included three glucosidases—GANAB, MOGS, and PRKCSH—which were highly correlated to high mannose-containing IGPs, but negatively correlated to more complex type glycans (Fig. 6a). The second group included the three subunits of the OST complex (DDOST, RPN1, and RPN2), which binds to the membrane-anchored Dol-P-P-oligosaccharide and transfers the glycan to the nascent protein. Interestingly, this latter group showed no clear differences in correlation between high mannose and complex types. Investigation of individual glycosylation enzymes revealed the glycosidase MAN1A1 was negatively correlated to intact glycopeptides carrying high mannose structures (Fig. 6b). Similarly, the fucosidases, FUCA1 and FUCA2, were found to have a negative correlation to fucosylated glycopeptides, whereas the fucosyltransferase, FUT11, showed a positive correlation to the fucosylated peptides. Moreover, sialylation-related enzymes, ST3GAL1 and ST6GALNAC, correlate to sialylated IGPs. We found mRNA expression data to be a poor correlator of measured known enzyme-glycan activity shown in Fig. 6b (Supplementary Fig. 6A and B). This feature was best exemplified by the relationship of the glucosidase PRKCSH and high mannose-containing IGPs. PRKCSH is the glucosidase that trims glucose residues from the immature glycan precursor in the endoplasmic reticulum, and although global protein levels of PRKCSH correlated with high mannose-containing IGPs, mRNA-level expression was much more dynamic across all tumors with no observed relationship to IGP composition structure. Overall, these results indicate that the protein level of glycosylation enzymes is more reflective of glycosylation activity rather than mRNA level expression, and those glyco-related genes may undergo post-transcriptional regulation^33^.

### Glycoproteomics-based model of HGSC

**Fig. 7:**
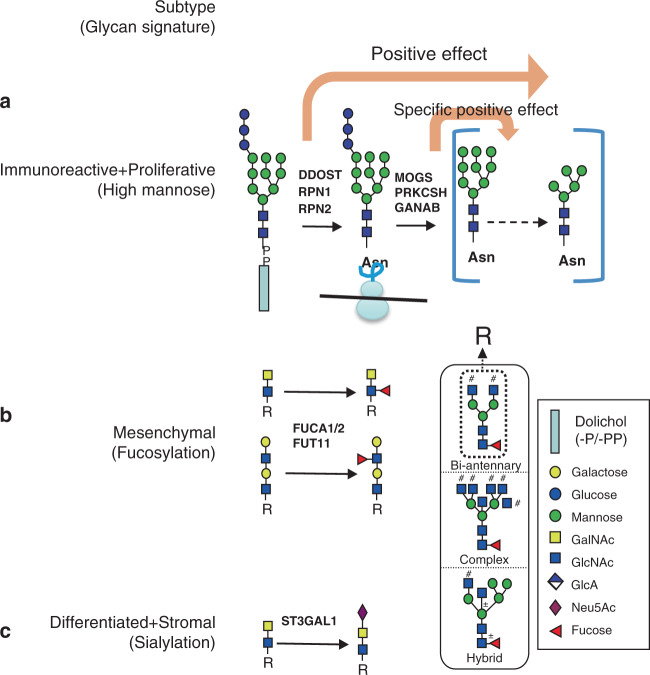
Glycoprocessing models of different HGSC subtypes. To summarize the molecular feature of ovarian cancer subtypes, we proposed three models involving glycans, glycosylation enzymes, and glycan biosynthesis pathways.

Based on the relationship among different glycans and glycosylation enzymes, we proposed glycoproteomic-based signatures regulated by different glycan biosynthesis pathways in the three HGSC tumor clusters (Fig. 7). For immunoreactive and proliferative subtypes represented by IGP cluster 2, high mannose is elevated, which may be impacted, in part, by the expression of glucosidases and certain subunits of OST complex. Glucosidases have a specific effect on high mannose, while the subunits of OST complex have a broad effect on N-linked glycan synthesis. For the mesenchymal subtype that was highly enriched, represented by IGP cluster 3, the down-regulation of fucosidases (FUCA1 and FUCA2) and up-regulation of fucosyltransferase, FUT11, contributed to the observed fucosylation pattern. While the differentiated and stromal subtypes were represented by IGP cluster 1 that was defined by sialylation and the two enzymes, ST3GAL1 and ST6GALNAC.

## Discussion

Significant inter-tumor heterogeneity was observed across human HGSC delineating molecular subtypes defined by transcriptomic and proteomic features^10,11^. In this study, we evaluated the contribution of glycosylation to disease heterogeneity in HGSC, revealing three major tumor clusters defined by intact glycopeptide patterns. Moreover, a deeper characterization of the prominent glycan composition of each of these three clusters delineated specific glycoforms associated with protein levels of glyco-enzyme expression and inferred activity. Integrating previous characterized proteomic subtype information showed select glycoform features were enriched, including the “Differentiated” and “Stromal” subtypes enriched in sialylated and fucosylated intact glycopeptides, the “Immunoreactive” and “Proliferative” subtypes enriched in high-mannose intact glycopeptides, and the “Mesenchymal” subtype enriched in fucosylated only intact glycopeptides. Overall, these results highlight the molecular complexity of HGSC tissues, and the distinct information that is provided at the transcriptional, translational, and post-translational levels. Many studies have focused on tumor subtyping of HGSC based on gene expression profiles. TCGA study using mRNA expression showed four transcriptomic HGSC subtypes defined as differentiated, immunoreactive, mesenchymal, and proliferative^10^. In CPTAC’s proteomic study, TCGA samples were designated as differentiated, immunoreactive, mesenchymal, proliferative, and stromal subtypes^11^. However, the role of glycosylation in tumor heterogeneity remains unknown. In this study, we have provided the most comprehensive glycoproteomic characterization of 119 HGSC samples to-date through measurements of glycosite-containing peptides (SPEGs) and intact glycopeptides (IGPs).

To gain insight into the functional relationship of our observed glycoform profiles, our protein–protein interaction analysis showed fucosylated glycans were carried by extracellular matrix-associated proteins, sialylated glycans by proteins involved in complement cascades, and high mannose glycans by lysosomal proteins (Supplementary Fig. 3). Fucosylated glycoproteins are often expressed on cell surfaces, or secreted in biological fluids, and are involved in a variety of functions related to cell-to-cell adhesion and recognition processes^34,35^. Although sialic acid is well known to play a role in complement cascades, few studies focused on the function consequences of sialylated glycoproteins in this cellular process^36^. Finally, unlike the oligosaccharides on secretory and membrane N-glycoproteins, which are processed to complex-type glycoforms, mannose residues often function in conjunction with phosphorylation to localize lysosomal enzymes to the lysosome organelles^37^. Through survival analysis, fucosylated peptides could be used in survival prediction (Fig. 3). Studies have shown that elimination of terminal fucose with fucosidase or through the knockdown of fucosytransferase inhibits tumor growth^38,39^.

The attached glycan structures on the glycoprotein backbone reflect the concerted enzymatic activity of multiple glycosylation enzymes. Previously, it was demonstrated that these changes in glycosylation are dependent on biochemical factors, such as the availability of nucleotide sugar pools and differential expression of certain glycosyltransferases^40^. However, just how complex the role of aberrant glycosylation is in carcinogenesis is still unclear. To address this challenging question, we leveraged a multi-faceted approach investigating the degree of glycosylation disturbance, concordance of glyco-enzyme expression and glycoform abundance, and the regulatory elements of glyco-enzyme expression. We found global protein expression to be the primary factor for downstream observations related to glycoform expression. Genomic-associated and transcriptomic-associated events were found to have minimal influence on the observed glycosylation patterns. Previously, select microRNAs were reported to play crucial role in controlling the levels of specific glycosyltransferases involved in cancer^33^, and although post-transcriptional level regulation was not investigated in our study, future exploration is warranted.

Since glycosylation enzymes were associated with protein glycosylation when contributing to tumor heterogeneity, we further explored the potential role of glyco-related genes in the production of specific glycans. The synthesis of N-linked glycans is initiated by the synthesis of a Glc3Man9GlcNAc2 lipid-linked precursor and the co-translational transfer to nascent polypeptide chains in the lumen of the endoplasmic reticulum (ER). Immediately after transfer of the glycan to Asn side chains glucose (Glc), trimming is initiated by the cleavage of the terminal a1,2-Glc residue by a-glucosidase I (MOGS)^41^. Subsequent cleavage of the two internal a1,3-Glc residues is accomplished by the heterodimeric enzyme a-glucosidase II (GANAB/PRKCSH)^42,43^ to produce the Man9GlcNAc2-Asn processing intermediate (Supplementary Fig. 5). The intermediate could be further trimmed by mannosidase, like mannosyl-oligosaccharide 1,2-alpha-mannosidase IA (MAN1A1). Reduced mannosidase MAN1A1 expression leads to aberrant N-glycosylation and impaired survival in breast cancer^44^. All three glucosidases (MOGS/GANAB/PRKCSH) show significant positive correlation to high mannose glycopeptides, whereas MAN1A1 shows negative correlation in this study (Fig. 6a and b). The turnover of fucose residues in glycoconjugates is achieved through the involvement not only of fucosyltransferases, but also of the fucosidase. Alpha-l-fucosidase, encoded by two genes—FUCA1, which codes the tissue enzyme^45^, and FUCA2, which leads to plasma alpha-l-fucosidase^46^—is a lysosomal enzyme that removes terminal l-fucose residues present on the oligosaccharide chains of glycoconjugates. Inhibiting the ER α-glucosidases was proposed to be used in treating viral infections^21^, and could also be considered as an approach to ovarian cancer treatment. FUCA1 and FUCA2 show negative correlation whereas fucosyltransferase 11 (FUT11) shows positive correlations to the product of fucosylated peptides. For sialylation, ST6 beta-galactoside alpha-2,6-sialyltransferase 1 (ST6GAL1) is a well-known N-glycan sialyltransferase^47^, but we did not observe a strong correlation between ST6GAL1 and sialylated peptide. However, two O-glycan sialyltransferases, ST3 β-galactoside α-2,3-sialyltransferase 1 (ST3GAL1)^48^, and ST6 (α-N-acetyl-neuraminyl-2,3-β-galac tosyl-1,3)-N-acetylgalactosaminide α-2,6-sialyltransferase 1 (ST6GALNac1)^49^, show strong correlations to sialylated peptides. ST3GAL1 was found to have several conserved features with ST6GAL1^50^. Whether these two O-glycan sialyltransferases have an effect on N-glycan peptides has yet to be confirmed. In addition, Tumor suppressor candidate 3 (TUSC3) is also correlated with sialylated peptide (Fig. 6b). The previous study revealed an increase in sialylation in TUSC3-positive H134 ovarian cancer cell lines compared to TUSC3-negative cells^51^. That information could help us to control the biosynthesis of glycosylated proteins. As a result, we have proposed three models for three glyco clusters of HGSC (Fig. 7). For immunoreactive and proliferative subtypes, OST complex may take a broadly positive effect, whereas glucosidases have a specific effect on the production of glycopeptides carrying high mannose. A mesenchymal subtype is mainly related to fucosylation, fucosidases, and FUT11 expression. Two sialyltransferases (ST3GAL1 and ST6GALNAC1) may be related to differential and stromal subtypes. That information could provide some clues for gene therapy targeted on glycosylation genes. Several drugs have been used for targeting glycosylation genes; for example, castanopermine inhibits glucosidase I and leads to altered glycoproteins with Glc3Man7GlcNAc2 structures^52^. Interestingly, mRNA expression of some glycosylation genes does not correlate well to those intact glycopeptides, which may have been caused by posttranscriptional regulation of those glyco-related genes.

In conclusion, our results show that glycosylation further contributes to the observed heterogeneity of HGSC tumors, with specific glycoform features associated with disease progression and severity. Integration of our comprehensive profiling of the N-linked glycoproteome with preexisting datasets characterizing genomic, transcriptomic, and proteomic features of HGSC reveals the unique insights that are gained by exploring this prominent post-translational modification. Glycoproteomic study identified that glycans conjugated on glycosites are associated with tumor subtypes and intact glycopeptides from a combination of glycosylation sites and site-specific glycosylation provides a survival predictor beyond proteins and transcripts of the glycoproteins. Furthermore, integration of glycoproteomics and global proteomics revealed that glycan biosynthesis in tumor subtypes might be controlled by glycan processing enzymes, and are further indicative to clinical outcomes. Overall, our study will serve as a comprehensive resource to the community in the areas of ovarian oncology and glycobiology, and will contribute to leveraging glycoform information for clinical diagnostic applications and therapeutic intervention.

## Methods

### Tumor samples

The tumor samples used in this manuscript are from the TCGA Biospecimen Core Resource, as described previously^11^. Demographics, histopathologic information, and treatment details were collected^11^. Clinical-pathologic characteristics of TCGA ovarian cases selected for glycoproteomic analysis are summarized in Supplementary Data 1. The average age at diagnosis was 59.9 years. We have complied with all relevant ethical regulations and obtained informed consent from all participants; Johns Hopkins University approved the study protocol.

### Protein extraction and tryptic digestion

Approximately 50 mg of each of the sectioned TCGA ovarian tumor tissues were sonicated separately in 1.5 mL of lysis buffer (8 M urea, 0.8 M NH_4_HCO_3_, pH 8.0). The protein concentrations of the lysates were determined by BCA assay (Pierce). Proteins were reduced with 10 mM TCEP for 1 h at 37 °C, and subsequently alkylated with 12 mM iodoacetamide for 1 h at RT in the dark. Samples were diluted 1:4 with deionized water and digested with sequencing grade modified trypsin at a 1:50 enzyme‐to‐protein ratio. After 12 h of digestion at 37 °C, another aliquot of the same amount of trypsin was added to the samples and further incubated at 37 °C overnight. The digested samples were then acidified with 10% trifluoroacetic acid to pH < 3. Tryptic peptides were desalted on C18 columns and dried using Speed-Vac.

### iTRAQ labeling of peptides

Desalted peptides were labeled with 4-plex iTRAQ reagents according to the manufacturer’s instructions (AB Sciex, Foster City, CA). Peptides (1 mg) from one pooled tissue, the reference sample, and three TCGA tumors were dissolved in 125 μL of 0.5 M triethylammonium bicarbonate, pH 8.5 solution, and further mixed with 5 units of iTRAQ reagent that was dissolved freshly in 375 μL of ethanol. Channel 114 was used for labeling the pooled reference sample throughout the TCGA sample analysis. After 2 h of incubation at room temperature, 10% TFA solution was added to bring it to a pH < 3 to stop the reaction. Peptides labeled by different iTRAQ reagents were then mixed and 200 μg of iTRAQ-labeled peptides were desalted on strong cation exchange columns and fractionated by offline bRPLC for global proteomic analysis^11^.

### Enrichment of glycosite-containing peptides using SPEG

Labeled peptides (3.6 mg) were oxidized in 60% ACN/0.1% TFA by a 10 mM NaIO_4_ solution at room temperature for 1 h in the dark. The samples were desalted by a C18 SPE column and the elution solution was collected into equilibrated hydrazide resin (180 μL of 50% slurry for each sample) directly and incubated with 100 mM aniline at room temperature overnight with shaking. The resin was washed three times each with 1 mL of 50% ACN, 1.5 M NaCl, water, and 25 mM NH_4_HCO_3_ buffer. N-glycopeptides were released via 3 μL PNGase F (New England Biolabs, Beverly, MA) in 25 mM NH_4_HCO_3_ buffer at 37 °C overnight with shaking. *N*-linked glycosite-containing peptides were collected in supernatants/wash solutions and dried by vacuum. The glycosite-containing peptides were resuspended in 50 μL 0.2% FA solution for LC–MS/MS analysis.

### Enrichment of intact glycopeptides by Retain AX Cartridges (RAX)

The remainder of the 200 μg of 4-plex iTRAQ-labeled peptides from each set were adjusted to 95% ACN (v/v), 1% TFA (v/v) for intact glycopeptide enrichment using RAX (particle size 30–50 μm, 30 mg sorbent per cartridge, Thermo Fisher Scientific). The RAX columns were equilibrated three times with 1 mL of ACN, three times with 100 mM triethylammonium acetate, three times with water, and finally three times with 95% ACN (v/v), 1% TFA (v/v). The samples were loaded onto RAX columns and washed four times with 1 mL of 95% ACN, 1% TFA. Finally, bound intact glycopeptides were eluted in 400 μL of 50% ACN (v/v), 0.1% TFA (v/v). The intact glycopeptides were then dried in a Speed-Vac and stored at −80 °C prior to LC–MS/MS analysis.

### LC–MS/MS for glycoproteomic analysis

The de-glycosylated glycosite-containing peptides were separated on a Dionex Ultimate 3000 RSLC nano system (Thermo Scientific) with a 75 µm × 50 cm Acclaim PepMap RSLC C18 Easy-Spray column (Thermo Scientific) protected by a 100 μm × 2 cm Acclaim PepMap 100 guard column (Thermo Scientific). Mobile phase flow rate was 320 nL/min and consisted of 0.1% formic acid in water (A) and 0.1% formic acid 95% acetonitrile (B). The sample injected (6 μL) was trapped using 100% mobile phase A for 13 min at a flow rate of 5 μL/min before being placed in-line with the analytical column and subjected to the gradient profile which was set as follows: 2–7% B for 10 min, 7–27% B for 80 min, 27–34% B for 22 min, 34–95% B for 3 min, and 95% B for 10 min. MS analysis was performed using a Q-Exactive mass spectrometer (Thermo Scientific). The Q-Exactive mass spectrometer parameters were as follows: electrospray voltage was 2.2 kV; following a 20 min delay from the end of sample trapping, Orbitrap precursor spectra (AGC 3 × 10^6^) were collected from 400 to 1800*m*/*z* for 110 min at a resolution of 70K along with the top 12 data-dependent Orbitrap HCD MS/MS spectra at a resolution of 35K (AGC 2 × 10^5^) and max ion time of 120 ms; ions selected for MS/MS were isolated at a width of 1.4*m*/*z* and fragmented using a normalized collision energy of 31%; peptide match was set to ‘Preferred’; exclude isotopes was set to ‘on’; and charge state screening was enabled to reject unassigned 1+, and >8+ ions with a dynamic exclusion time of 30 s to discriminate against previously analyzed ions. Each sample was analyzed by LC–MS/MS in triplicates to increase the SPEG coverages and reduce the missing values, which is similar to fractionation in proteomics experiments^53^.

The intact glycopeptides were analyzed on the Orbitrap Fusion Lumos system (Thermo Scientific). The glycopeptides were separated using Easy nLC 1200 UHPLC system (Thermo Scientific) on an in-house packed 20 cm × 75 mm diameter C18 column (1.9 mm Reprosil-Pur C18-AQ beads, Dr. Maisch GmbH); Picofrit 10 mm opening (New Objective). The column was heated to 50 °C using a column heater (Phoenix-ST). The flow rate was 0.200 μl/min with 0.1% formic acid and 2% acetonitrile in water (A) and 0.1% formic acid, 90% acetonitrile (B). Peptides injected were subjected to the following gradient: 2–6% B for 1 min, 6–30% B for 84 min, 30–60% B for 9 min, 60–90% B for 1 min, 90% B for 5 min, and then back to 50% B for 10 min. The Fusion Lumos mass spectrometer parameters were as follows: electrospray voltage was 1.8 kV; the ion transfer tube temperature was at 250 °C; Orbitrap precursor spectra (AGC 4 × 10^5^) were collected from 350–1800*m*/*z* for 110 min at a resolution of 60K along with data-dependent Orbitrap HCD MS/MS spectra (centroided) at a resolution of 50K (AGC 2 × 10^5^) and max ion time of 105 ms for a total duty cycle of 2 s; masses selected for MS/MS were isolated (quadrupole) at a width of 0.7*m*/*z* and fragmented using a high-energy collision dissociation of 38%; peptide charge state screening was enabled to reject unassigned 1+, 7+, 8+, and >8+ ions with a dynamic exclusion time of 45 s to discriminate against previously analyzed ions between ±10 ppm. Similarly, each sample was analyzed by LC–MS/MS in triplicates to increase the IGP coverage and reduce the missing values^53^.

### Quantification of glycosite-containing peptides from SPEG

MS-PyCloud, version 1.5.0, was used to identify and quantify glycosite-containing peptides from SPEG^54^. RAW files were first converted to universal mzML format using ProteoWizard 3.0^55^. NCBI RefSeq human protein fasta database retrieved on 2 May 2016 was utilized for the database search. MS-GF+ search engine was then used to assign spectra, within a precursor mass tolerance of 20 ppm, with the following modifications: dynamic deamidation (+0.984016 Da) on Asn and Gln, static oxidation (+15.9949 Da) on Met, static iTRAQ4plex (+144.102063 Da) on peptide N-terminus and Lys, and static carbamidomethylation (+57.021464 Da) on Cys. Peptides were then quantified and assigned to protein groups using the following filters: a minimum requirement of 1 PSM/peptide after filtering PSMs by the −Log10(MS-GF+ SpectralEValue) score for each charge state separately such that the PSM-level FDR remained below 1%. Two Trypsin cleavage termini were required, along with an allowance of up to two missed cleavages. The rule of NXS/T (X can be any amino acid except P) motif on peptide sequence for N-glycosylation site was applied to verify the identified peptides. For SPEG dataset, the quantitative data was summarized not by the protein-level, but rather at the glycosite-containing peptide level (Supplementary Data 2).

All the peptides (intact glycopeptides, glycosite-containing peptides, and global peptides) were labeled with iTRAQ reagent simultaneously. Separation for glycosite-containing peptide and intact glycopeptide analysis was performed after the labeling. Thus, the labeling is identical among global proteomic, glycosite-containing peptide and intact glycopeptide datasets. Therefore, for glycosite-containing peptide analysis, we applied the normalization factors in the global proteomic dataset to normalize the relative abundance of glycosite-containing peptides.

### Quantification of intact *N*-linked glycopeptides

The intact *N*-linked glycopeptides were identified using GPQuest 2.0 software^56^. Prior to search, Proteowizard 3.0 was used to convert the.RAW files to.mzML files with the “centroid all MS2 scans” option selected^55^. GPQuest 2.0 was applied to investigate the expression of protein glycosylation on the unidentified MS/MS spectra using two approaches: the searching of spectra containing oxonium ions (‘oxo-spectra’) and the identification of intact *N-*linked glycopeptides. The oxonium ions were regarded as the signature features of the glycopeptides assigned to the MS/MS spectra, which were caused by the fragmentation of glycans attached to intact glycopeptides in the mass spectrometer. In this study, the MS/MS spectra containing the oxonium ions (*m*/*z* 204.0966) in the top 10 abundant peaks after removing reporter ions were considered to be potential glycopeptide candidates. The intact *N-*linked glycopeptides were identified by using GPQuest 2.0 to search against the database of glycosite-containing peptides identified from the SPEG dataset from this study and a database containing 178 *N-*linked glycan compositions. The glycan database was collected from the public database of GlycomeDB^57^ (http://www.glycome-db.org). All the qualified (>6 fragment ions matchings) candidate peptides were compared with the spectrum again to calculate the Morpheus scores by considering all the peptide fragments, glycopeptide fragments, and their isotope peaks. The peptide having highest Morpheus score was then assigned to the spectrum. The mass gap between the assigned peptide and the precursor mass was searched in the glycan database to find the associated glycan. The best hits of all “oxo-spectra” were ranked by Morpheus score in descending order, for which those with a FDR < 1% and covering >10% total intensity of each tandem spectrum were reserved as qualified identifications. The precursor mass tolerance was set as 10 ppm, and the fragment mass tolerance was 20 ppm. Similar to the process of glycosite-containing peptides, the quantification of the intact glycopeptides was also determined at the peptide level. The median log2 ratio value of all the PSMs of an identical intact glycopeptide was used as the relative abundance of the intact glycopeptide. The relative abundances of intact glycopeptides of samples were also normalized using the median value of glycoproteins quantified in the global datasets.

### Glycoproteomic subtyping analysis

The glycopeptides and intact glycopeptides without missing values were analyzed by CancerSubtypes^58^ for consensus clustering of tumor subtypes. Specifically, 80% of the original sample pool was randomly subsampled without replacement and partitioned into three major clusters using partitioning around medoids (PAM) algorithm, which was repeated 500 times. The expression values were transformed into *Z*-scores using the built-in standardization function of R 3.2.2. For the IGP clustering, five groups of intact glycopeptdes were the corresponding glycan types and were also listed on the left side of the heatmap of the clustered expression matrix to illustrate the possible relationship between tumor subtypes and the associated glycan types.

### Protein-glycopeptide expression consistency analysis

For each glycosite and intact glycopeptide without missing values, we calculated the Spearman correlation between glycosite abundance and its corresponding global protein abundance and mRNA gene expression across TCGA tumor samples, respectively. Correlation *p*-values were adjusted for multiple hypotheses testing using the Benjamini–Hochberg procedure.

### Protein–protein interaction network analysis

A group of interested genes was inputted in the STRING v11^31^ to perform protein–protein interaction network analysis. The proteins with enrichment terms were highlighted.

### Glycosylation biosynthetic pathway analysis

The intact glycopeptide expression was hypothesized to be influenced by at least the expression of two factors: substrates (glycoprotein precursors) and glycosylation enzymes. The log2 ratio values of intact glycopeptides were correlated with the 22 glycosylation enzymes identified from the global proteomic data. The correlation matrix was further arranged by hierarchical clustering on glycopeptides (columns) and glycosylation enzymes (rows) and visualized in Fig. 5a. The glycan compositions were linked to the intact glycopeptides. For each comparison, the correlations between the intact glycopeptides and specific glycosylation enzymes (FUT11, PRKCSH, or MAN1A1) across all samples were calculated and shown in a boxplot.

### Survival analysis on the intact glycoproteomics-based signature of the mesenchymal subtype

An index score was derived for each sample as the mean abundance of intact glycoproteins that defined the mesenchymal subtype on glycol proteomics data, global proteomics data, and mRNA gene expression data. We compared 50 samples with higher scores and 50 samples with lower scores for Kaplan–Meier survival analysis, and the log-rank *p*-value was calculated using the Lifelines python package (version 0.24.16)^59^. Cox proportional hazards regression analysis (including confidence intervals and *p*-values) were conducted by fitting the age and IGP clusters using the “CoxPHFitter” function.

### Statistical analysis

Statistical analyses, including a *t*-test and a hypergeometric test, were conducted using the statistical package Python (version 3.7.4) and R (version 3.2.2).

### Reporting summary

Further information on research design is available in the Nature Research Reporting Summary linked to this article.

## Supplementary information

Supplementary Information

Description of Additional Supplementary Files

Supplementary Data 1

Supplementary Data 2

Supplementary Data 3

Reporting Summary

## Source data

Source Data

## Data Availability

Raw data files and processed files of glycoproteomics data generated in this study have been deposited to the ProteomeXchange Consortium (http://www.proteomexchange.org/) through MassIVE (https://massive.ucsd.edu) with the accession codes “PXD019914” and “MSV000085613 [10.25345/C5VX3Q]”. Proteomics and genomics data were collected from the CPTAC Data Portal (https://cptacdcc.georgetown.edu/cptac/s?id=54784), and cBioPortal (https://www.cbioportal.org/), respectively. The glycan database was collected from the public database of GlycomeDB (http://www.glycome-db.org).  Source data are provided with this paper.
