# Supplementary material for: Glycoproteomics-based signatures for tumor subtyping and clinical outcome prediction of high-grade serous ovarian cancer
Source: Nat Commun. 2020 Dec 1;11:6139. doi: 10.1038/s41467-020-19976-3 (PMC7708455; doi:10.1038/s41467-020-19976-3)
Supplement: Supplementary file 1 — Supplementary Information [file 41467_2020_19976_MOESM1_ESM.pdf]

Supplementary Information

Integrated Proteomic and Glycoproteomic Characterization of Human High-Grade Serous Ovarian Carcinoma

Pan et al.

Supplementary Fig. 1

Schematic diagram of the workflow.

(a) MS-based glycoproteomics and proteomics of ovarian cancer samples. (b) Data analysis workflow.

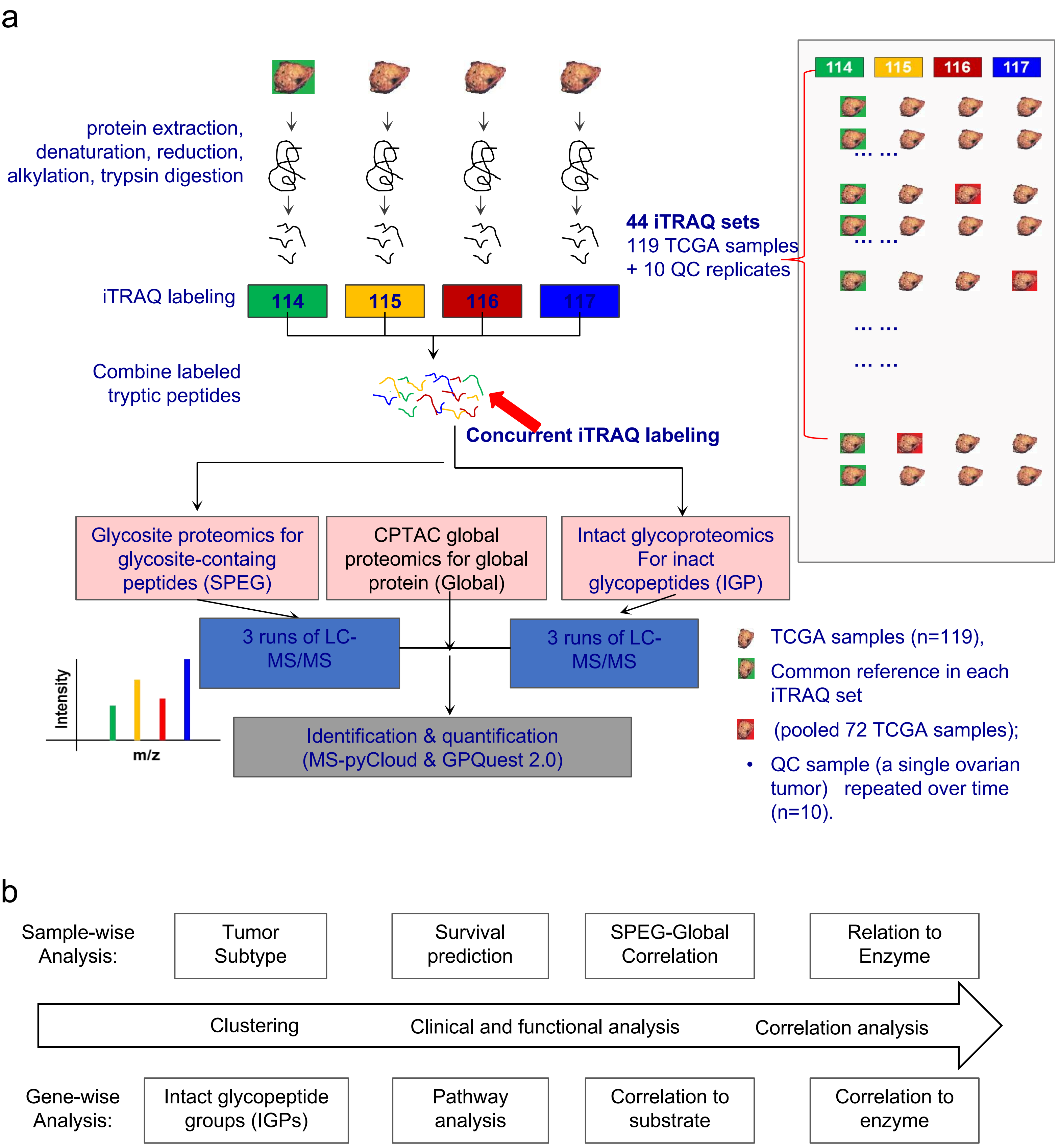
a
protein extraction, denaturation, reduction, alkylation, trypsin digestion
iTRAQ labeling
114
115
116
117
Combine labeled tryptic peptides
Concurrent iTRAQ labeling
44 iTRAQ sets
119 TCGA samples
+ 10 QC replicates
Glycosite proteomics for glycosite-containg peptides (SPEG)
CPTAC global proteomics for global protein (Global)
Intact glycoproteomics For inact glycopeptides (IGP)
3 runs of LC-MS/MS
3 runs of LC-MS/MS
Intensity
m/z
Identification & quantification (MS-pyCloud & GPQuest 2.0)
TCGA samples (n=119),
Common reference in each iTRAQ set
(pooled 72 TCGA samples);
QC sample (a single ovarian tumor) repeated over time (n=10).
b
Sample-wise Analysis:
Tumor Subtype
Survival prediction
SPEG-Global Correlation
Relation to Enzyme
Clustering
Clinical and functional analysis
Correlation analysis
Gene-wise Analysis:
Intact glycopeptide groups (IGPs)
Pathway analysis
Correlation to substrate
Correlation to enzyme

Supplementary Fig. 2

Quality control of glycoproteomic analysese of glycosite-containing peptides & intact glycopeptides.

(a) The distribution of coefficient of variation (CV) of glycosite-containing peptides of SPEG data. (b) The distribution of coefficient of variation (CV) of intact glycopeptides of IGP data. Source data are provided as a Source data file.

a

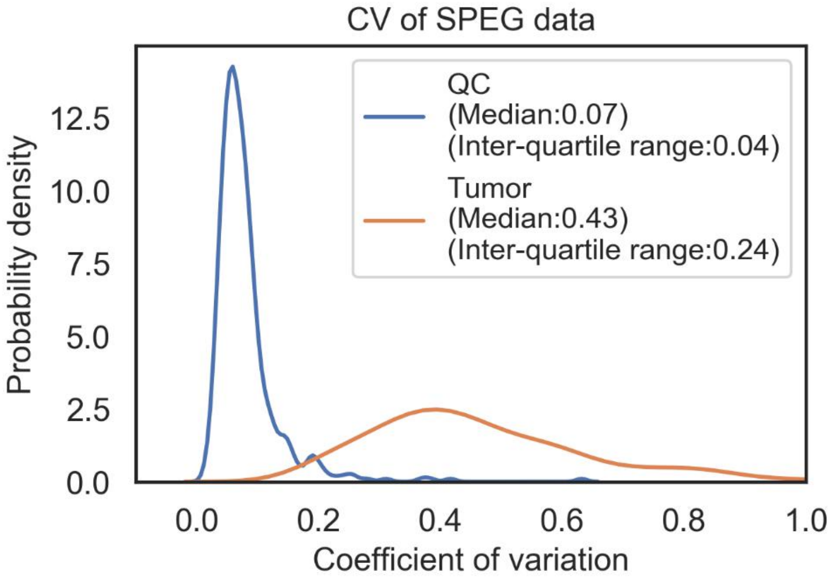
CV of SPEG data
QC
(Median:0.07)
(Inter-quartile range:0.04)
Tumor
(Median:0.43)
(Inter-quartile range:0.24)
Probability density
12.5
10.0
7.5
5.0
2.5
0.0
0.0
0.2
0.4
0.6
0.8
1.0
Coefficient of variation

b

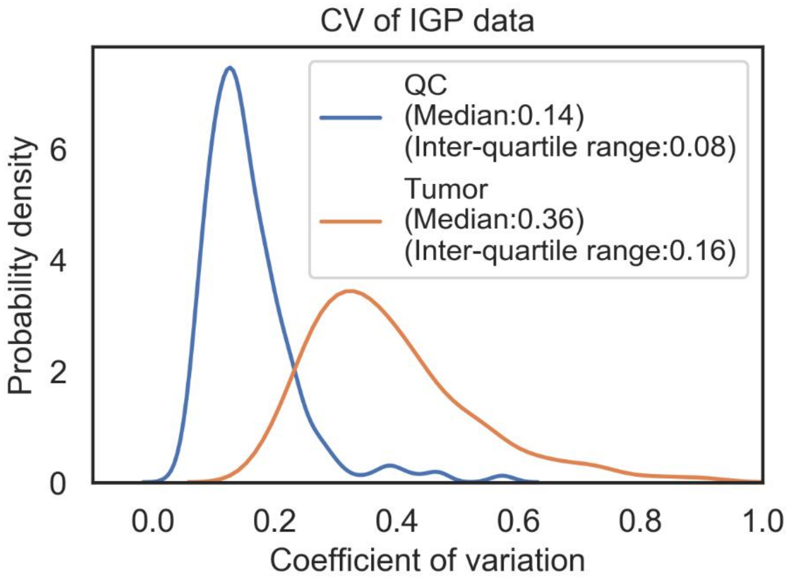
CV of IGP data
QC
(Median:0.14)
(Inter-quartile range:0.08)
Tumor
(Median:0.36)
(Inter-quartile range:0.16)
Probability density
6
4
2
0
0.0
0.2
0.4
0.6
0.8
1.0
Coefficient of variation

Supplementary Fig. 3

Protein-protein interaction network of glycoproteomics-based signatures

Group 1 (a), group 2 (b), group 3 (c), group 4 (d) and group 5 (e). FDR adjusted p value using a Hypergeometric test by STRING is provided for a-e.

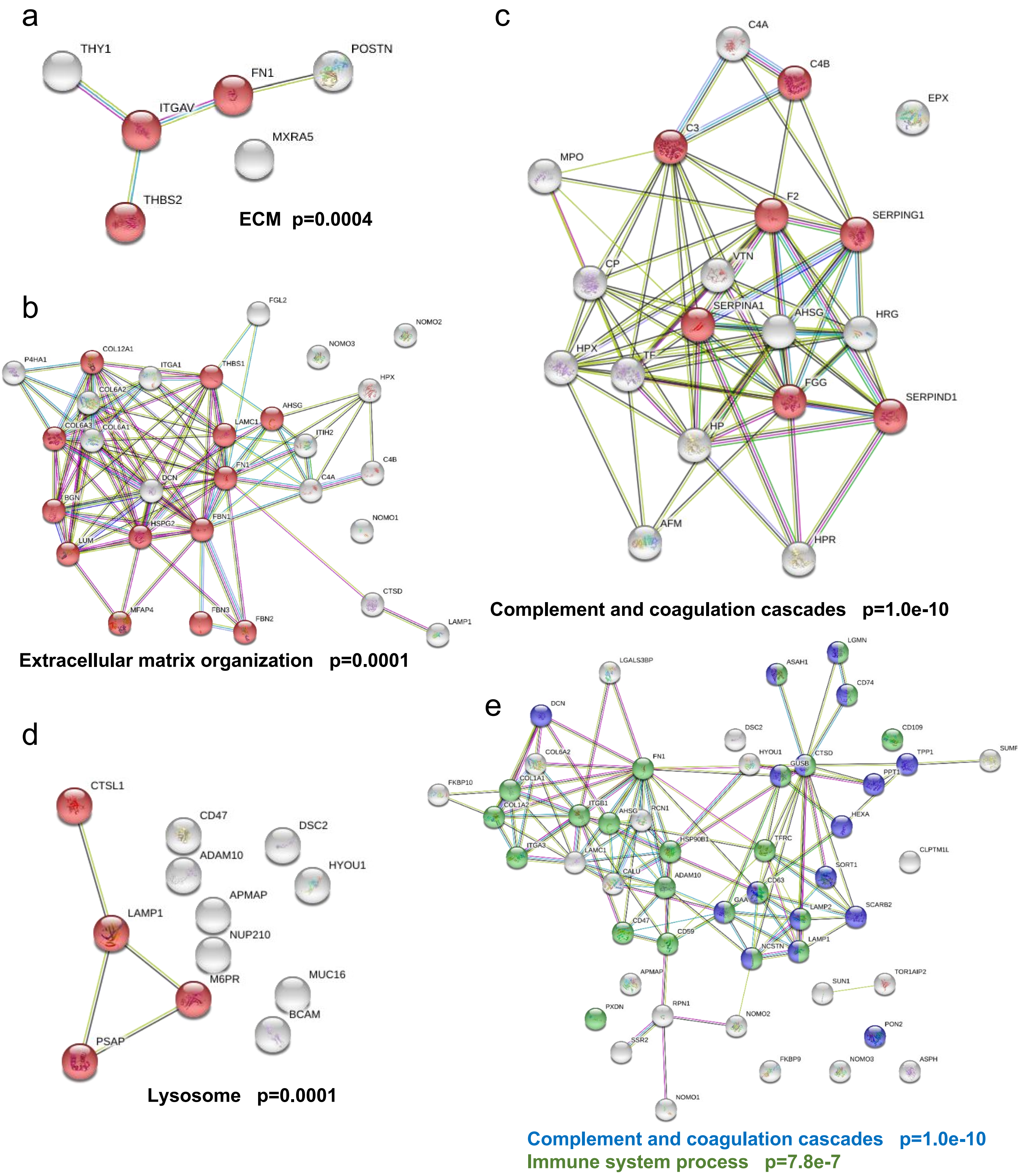
a
THY1
POSTN
FN1
ITGAV
MXRA5
THBS2
ECM p=0.0004
b
FGL2
NOMO2
NOMO3
P4HA1
COL12A1
ITGA1
THBS1
HPX
COL6A2
AHSG
COL6A3
COL6A1
LAMC1
ITIH2
C4B
FN1
DCN
C4A
BGN
FBN1
NOMO1
HSPG2
LUM
CTSD
MFAP4
FBN3
FBN2
LAMP1
Extracellular matrix organization p=0.0001
c
C4A
C4B
EPX
C3
MPO
F2
SERPING1
CP
VTN
SERPINA1
AHSG
HRG
HPX
TF
FGG
SERPIND1
HP
AFM
HPR
Complement and coagulation cascades p=1.0e-10
d
CTSL1
CD47
DSC2
ADAM10
HYOU1
APMAP
LAMP1
NUP210
M6PR
MUC16
BCAM
PSAP
Lysosome p=0.0001
e
LGMN
LGALS3BP
ASAH1
CD74
DCN
CD109
DSC2
COL6A2
FN1
HYOU1
CTSD
TPP1
SUMF
GUSB
PPT1
FKBP10
COL1A1
COL1A2
ITGB1
AHSG
RCN1
HEXA
HSP90B1
TFRC
ITGA3
LAMC1
CLPTM1L
SORT1
CALU
ADAM10
CD63
GAA
SCARB2
LAMP2
CD47
CD59
NCSTN
LAMP1
APMAP
SUN1
TOR1AIP2
RPN1
PXDN
NOMO2
PON2
SSR2
FKBP9
NOMO3
ASPH
NOMO1
Complement and coagulation cascades p=1.0e-10
Immune system process p=7.8e-7

Supplementary Fig. 4

Kaplan-Meier Plot of Overall Survival Stratified by IGP clusters or IGP group signatures.

(a) Group 1 signatures adjusted by age and IGP clusters. (b) Protein signatures of group 1. (c) mRNA signatures of group 1. (d) Group 3 signatures adjusted by age and IGP clusters. (e) Protein signatures of group 3. (f) mRNA signatures of group 3 (g) Group 4 signatures adjusted by age and IGP clusters. (h) Protein signatures of group 4. (i) mRNA signatures of group 4. In a-i, for both groups with highest scores and lowest scores, n = 50 samples. Logrank_test is used in a-i.

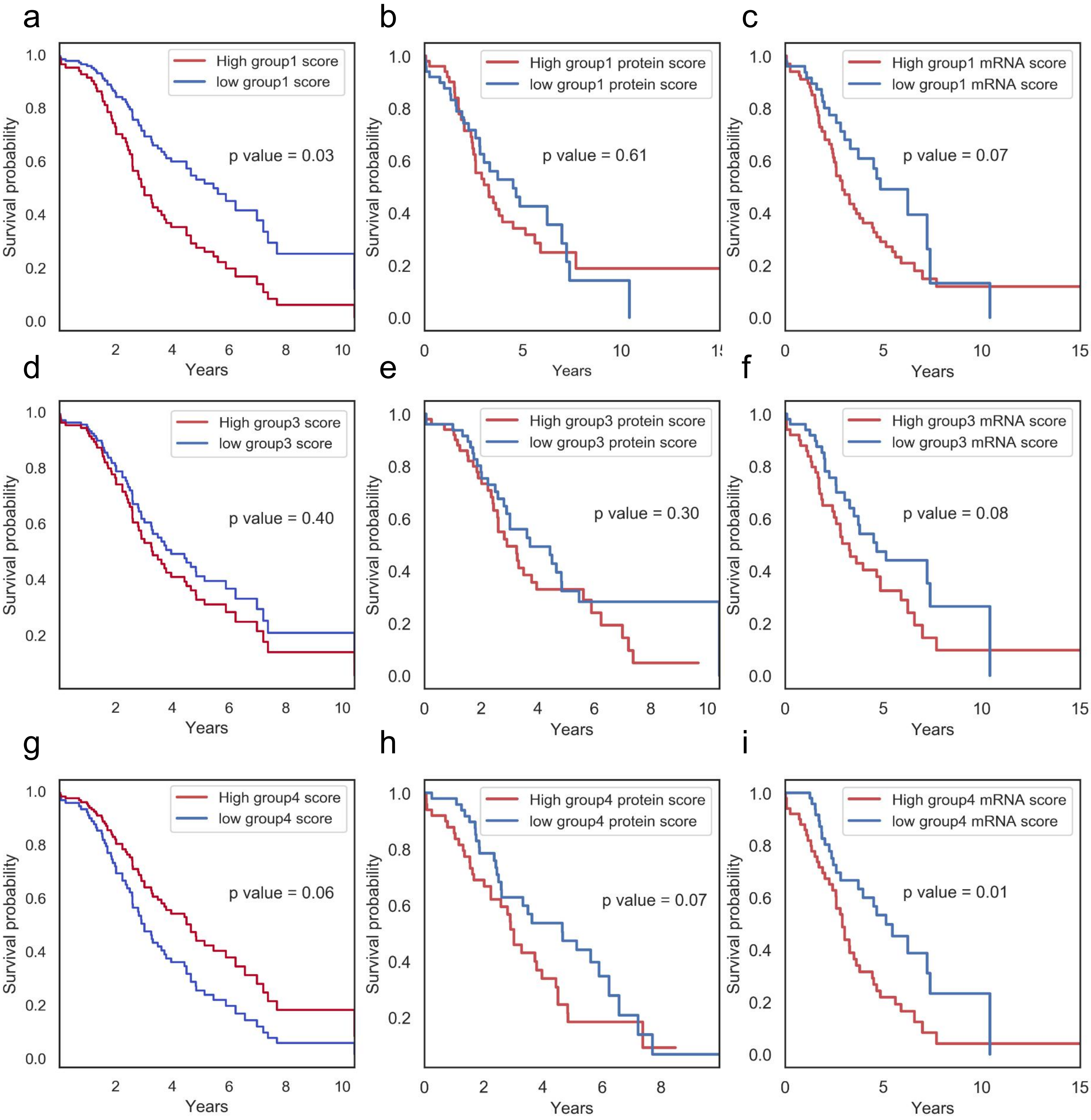

a
High group1 score
low group1 score
p value = 0.03
Survival probability
Years
b
High group1 protein score
low group1 protein score
p value = 0.61
Survival probability
Years
c
High group1 mRNA score
low group1 mRNA score
p value = 0.07
Survival probability
Years
d
High group3 score
low group3 score
p value = 0.40
Survival probability
Years
e
High group3 protein score
low group3 protein score
p value = 0.30
Survival probability
Years
f
High group3 mRNA score
low group3 mRNA score
p value = 0.08
Survival probability
Years
g
High group4 score
low group4 score
p value = 0.06
Survival probability
Years
h
High group4 protein score
low group4 protein score
p value = 0.07
Survival probability
Years
i
High group4 mRNA score
low group4 mRNA score
p value = 0.01
Survival probability
Years

Supplementary Fig. 5

N-linked glycosylation biosynthesis pathway.

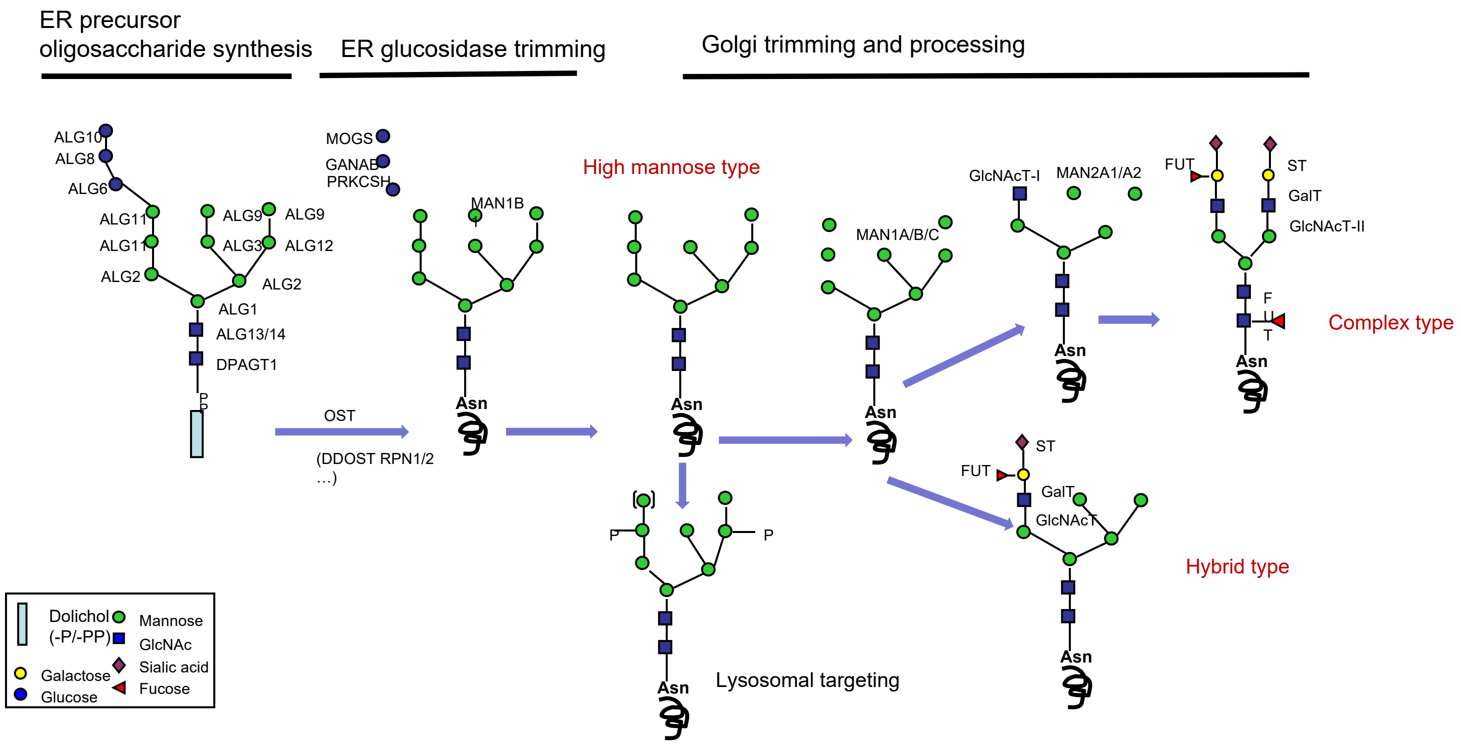

ER precursor oligosaccharide synthesis
ER glucosidase trimming
Golgi trimming and processing
ALG10
ALG8
ALG6
ALG11
ALG11
ALG2
ALG9
ALG3
ALG9
ALG12
ALG2
ALG1
ALG13/14
DPAGT1
P
OST
(DDOST RPN1/2 …)
MOGS
GANAB
PRKCSH
MAN1B
Asn
High mannose type
Asn
MAN1A/B/C
Asn
P
P
Asn
Lysosomal targeting
GlcNAcT-I
MAN2A1/A2
Asn
FUT
ST
GalT
GlcNAcT-II
F
U
T
Asn
Complex type
ST
FUT
GalT
GlcNAcT
Asn
Hybrid type
Dolichol (-P/-PP)
Mannose
GlcNAc
Galactose
Sialic acid
Glucose
Fucose

Supplementary Fig. 6

Heatmap of expression correlation between N-linked glycosylation enzymes with protein or mRNA data and intact glycopeptides with glycan structures.

(a) Heatmaps were performed on correlations between intact glycopeptide expression (column) and glycosylation enzyme expression (left: protein expression; right: mRNA expression in rows) across 119 tumor samples. The order of enzymes and intact glycopeptides were extracted in Fig 5. Red indicates positive correlation while blue indicates negative correlation. (b) Boxplot panel of the correlation difference between enzyme expression (mRNA level) and specific intact glycopeptides. For PRKCSH, n (No) = 128 glycopeptides, n (Yes)= 47 glycopeptides, p = 0.194 (Yes vs. No); for MAN1A1, n (No) = 128 glycopeptides, n (Yes)= 47 glycopeptides, p = 6.59e-5 (Yes vs. No); for FUCA1, n (No) = 97 glycopeptides, n (Yes)= 78 glycopeptides, p = 3.91e-3 (Yes vs. No); for ST3GAL1, n (No) = 117 glycopeptides, n (Yes)= 58 glycopeptides, p = 1.12e-4 (Yes vs. No).Two-tailed unpaired t-test used in b. For b, the box outlines denote the IQR, the solid line in the box denotes median correlation value, and the whiskers outside of the box extend to the minimum and maximum correlation value. Source data are provided as a Source data file.

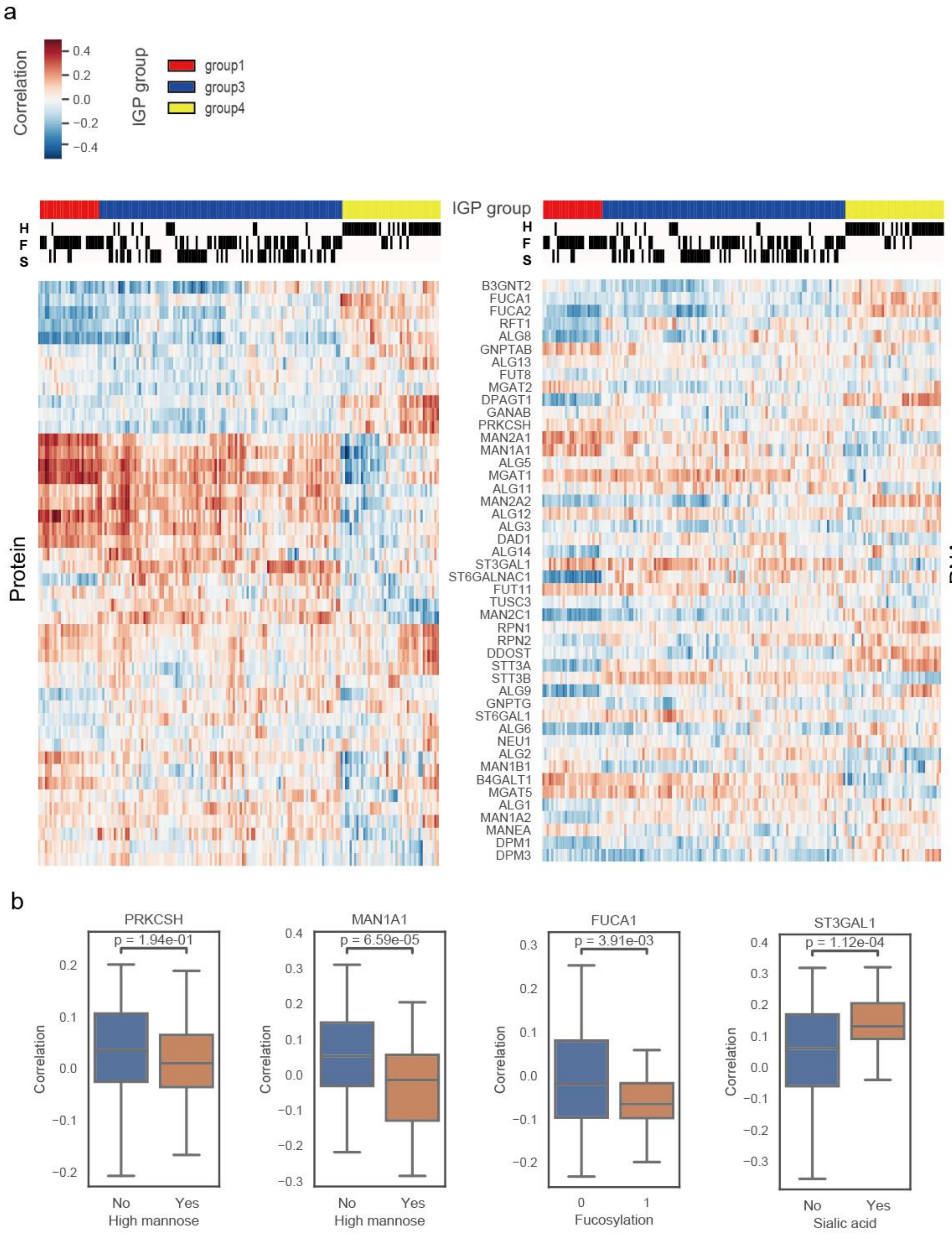
a
Correlation
0.4
0.2
0.0
−0.2
−0.4
IGP group
group1
group3
group4
IGP group
H
F
S
Protein
RNA
B3GNT2
FUCA1
FUCA2
RFT1
ALG8
GNPTAB
ALG13
FUT8
MGAT2
DPAGT1
GANAB
PRKCSH
MAN2A1
MAN1A1
ALG5
MGAT1
ALG11
MAN2A2
ALG12
ALG3
DAD1
ALG14
ST3GAL1
ST6GALNAC1
FUT11
TUSC3
MAN2C1
RPN1
RPN2
DDOST
STT3A
STT3B
ALG9
GNPTG
ST6GAL1
ALG6
NEU1
ALG2
MAN1B1
B4GALT1
MGAT5
ALG1
MAN1A2
MANEA
DPM1
DPM3
b
PRKCSH
p = 1.94e-01
Correlation
No
Yes
High mannose
MAN1A1
p = 6.59e-05
Correlation
No
Yes
High mannose
FUCA1
p = 3.91e-03
Correlation
0
1
Fucosylation
ST3GAL1
p = 1.12e-04
Correlation
No
Yes
Sialic acid
