# Supplementary material for: Glycoproteomics-based signatures for tumor subtyping and clinical outcome prediction of high-grade serous ovarian cancer
Source: Nat Commun. 2020 Dec 1;11:6139. doi: 10.1038/s41467-020-19976-3 (PMC7708455; doi:10.1038/s41467-020-19976-3)
Supplement: Supplementary file 2 — Description of Additional Supplementary Files [file 41467_2020_19976_MOESM2_ESM.docx]

**Description of Supplementary Files**

**File Name: Supplementary Data 1**

**Description:** Clinical sample information for glycoproteomic data.

**File Name: Supplementary Data 2**

**Description:**  Expression matrices of SPEG glycoproteomics (SPEGs) and intact glycoproteomics (IGPs). In the glyco motif column, “Y” indicates the presence of N-linked glycosylation motif (NxS/T) in the peptide, while “N” indicates no motif in the peptide. The “group” column indicates the information of intact glycopeptide group in Figure 1.

**File Name: Supplementary Data 3**

**Description:** Tumor subtype information and associated groups of intact glycopeptides.
