# Supplementary material for: Glycoproteomics-based signatures for tumor subtyping and clinical outcome prediction of high-grade serous ovarian cancer
Source: Nat Commun. 2020 Dec 1;11:6139. doi: 10.1038/s41467-020-19976-3 (PMC7708455; doi:10.1038/s41467-020-19976-3)
Supplement: Supplementary file 6 — Reporting Summary [file 41467_2020_19976_MOESM6_ESM.pdf]

nature research

Corresponding author(s): Hui Zhang

Last updated by author(s): Oct 22, 2020

Reporting Summary

Nature Research wishes to improve the reproducibility of the work that we publish. This form provides structure for consistency and transparency in reporting. For further information on Nature Research policies, see Authors & Referees and the Editorial Policy Checklist .

Statistics

For all statistical analyses, confirm that the following items are present in the figure legend, table legend, main text, or Methods section.

n/a	Confirmed	
☐	☒	The exact sample size (n) for each experimental group/condition, given as a discrete number and unit of measurement
☐	☒	A statement on whether measurements were taken from distinct samples or whether the same sample was measured repeatedly
☐	☒	The statistical test(s) used AND whether they are one- or two-sided<br>*Only common tests should be described solely by name; describe more complex techniques in the Methods section.*
☒	☐	A description of all covariates tested
☐	☒	A description of any assumptions or corrections, such as tests of normality and adjustment for multiple comparisons
☐	☒	A full description of the statistical parameters including central tendency (e.g. means) or other basic estimates (e.g. regression coefficient) AND variation (e.g. standard deviation) or associated estimates of uncertainty (e.g. confidence intervals)
☐	☒	For null hypothesis testing, the test statistic (e.g. F, t, r) with confidence intervals, effect sizes, degrees of freedom and P value noted<br>*Give P values as exact values whenever suitable.*
☒	☐	For Bayesian analysis, information on the choice of priors and Markov chain Monte Carlo settings
☐	☒	For hierarchical and complex designs, identification of the appropriate level for tests and full reporting of outcomes
☒	☐	Estimates of effect sizes (e.g. Cohen's d, Pearson's r), indicating how they were calculated

Our web collection on statistics for biologists contains articles on many of the points above.

Software and code

Policy information about availability of computer code

Data collection	The glycoproteomics analysis was performed using tandem mass spectrometry (LC-MS/MS)
Data analysis	The Glycosite-containing peptides were identified using PyCloud 1.5.0 and ProteoWizard 3.0. The intact N-linked glycopeptides were identified using GPQuest 2.0 and ProteoWizard 3.0 software. Python 3.7.4 , STRING v11, CancerSubtypes, R 3.2.2 and Lifelines python package (version 0.24.16) were used to do the downstream analysis.

For manuscripts utilizing custom algorithms or software that are central to the research but not yet described in published literature, software must be made available to editors/reviewers. We strongly encourage code deposition in a community repository (e.g. GitHub). See the Nature Research guidelines for submitting code & software for further information.

Data

Policy information about availability of data

All manuscripts must include a data availability statement. This statement should provide the following information, where applicable:

- Accession codes, unique identifiers, or web links for publicly available datasets
- A list of figures that have associated raw data
- A description of any restrictions on data availability

Raw data files and processed files of glycoproteomics data generated in this study have been deposited to the ProteomeXchange Consortium (http://www.proteomexchange.org/, Accession: PXD019914) through MassIVE (https://massive.ucsd.edu, Accession: MSV000085613). Proteomics and genomics data are collected from the CPTAC Data Portal (https://cptacdcc.georgetown.edu/cptac/s?id=54784), and cBioPortal (https://www.cbioportal.org/), respectively. The glycan database was collected from the public database of GlycomeDB (http://www.glycome-db.org). The source data for Figs. 2b,c, d, 4b, 5a, b, 6b and Supplementary Figs. S2, S6b are provided as a Source Data file.

Field-specific reporting

Please select the one below that is the best fit for your research. If you are not sure, read the appropriate sections before making your selection.

- [x] Life sciences
- [ ] Behavioural & social sciences
- [ ] Ecological, evolutionary & environmental sciences

For a reference copy of the document with all sections, see nature.com/documents/nr-reporting-summary-flat.pdf

Life sciences study design

All studies must disclose on these points even when the disclosure is negative.

Sample size	All of the 119 High-grade serous carcinoma samples from CPTAC ovarian proteomics cell paper were used in this study.
Data exclusions	No data exclusions
Replication	10 quality control samples were successfully measured for reproducibility.
Randomization	Not applicable for this study
Blinding	Not applicable for this study

Reporting for specific materials, systems and methods

We require information from authors about some types of materials, experimental systems and methods used in many studies. Here, indicate whether each material, system or method listed is relevant to your study. If you are not sure if a list item applies to your research, read the appropriate section before selecting a response.

Materials & experimental systems

n/a	Involved in the study	
x		Antibodies
x		Eukaryotic cell lines
x		Palaeontology
x		Animals and other organisms
	x	Human research participants
x		Clinical data

Methods

n/a	Involved in the study	
x		ChIP-seq
x		Flow cytometry
x		MRI-based neuroimaging

Human research participants

Policy information about studies involving human research participants

Population characteristics	Population characteristics was shown in supplementary table 1.
Recruitment	Not applicable for this study
Ethics oversight	We have complied with all relevant ethical regulations and obtained informed consent from all participants; Johns Hopkins University approved the study protocol.

Note that full information on the approval of the study protocol must also be provided in the manuscript.
